# Searching for peripheral proteomic markers of primary aldosteronism

**DOI:** 10.1007/s12020-025-04302-y

**Published:** 2025-06-13

**Authors:** Nikita Makhnov, Fredrik Axling, Elham Barazeghi, Peter Stålberg, Tobias Åkerström, Per Hellman

**Affiliations:** 1 Department of Surgical Sciences, Uppsala University, Uppsala, Sweden; 2 Department of Surgery, Karlstad Central Hospital, Karlstad, Sweden; 3 Center for Clinical Research and Education, Region of Värmland, Karlstad, Sweden; 4 Department of Surgery, Uppsala University Hospital, Uppsala, Sweden

**Keywords:** Primary aldosteronism, Diagnostics, Proteomics, Proteomic markers, Hypertension, Machine learning

## Abstract

**Purpose:**

Primary aldosteronism (PA) is prevalent among hypertensive patients, and associated with worsened cardiovascular outcomes compared to essential hypertension (HT). Screening and diagnostics for PA are currently complicated and invasive, why new methods are needed. Unilateral PA (uPA) is best treated surgically, and bilateral PA (bPA) - medically. No validated proteomic diagnostic test has been found yet. Our aim was to explore proteomic markers in peripheral serum to discriminate between HT, PA, uPA and bPA.

**Methods:**

Eighty-eight hypertensive individuals were evaluated for PA, and diagnosed with HT (n = 30); bPA (n = 29); and uPA (n = 29). Serum samples from these study groups were analyzed by Olink® Explore 384 Cardiometabolic Panel. A machine learning model based on ridge logistic regression with a stratified 5-fold cross-validation was used to identify HT, PA, bPA and uPA.

**Results:**

In the study groups, 56 circulating proteins were significantly different, and some of them specifically: 4 between PA vs. HT; 3 between bPA vs. uPA; 1 between bPA vs. HT; 9 between uPA vs. HT; 1 between HT vs. bPA vs. uPA. Three proteins with strongest differentiation (Coagulation factor IX for PA vs. HT; dipeptidyl peptidase 4 for uPA vs. HT and bPA; heat shock protein B1 for bPA vs. uPA) were validated by enzyme-linked immunosorbent assay. Our machine learning model could successfully identify 95% of HT, bPA, and uPA samples.

**Conclusion:**

Serum protein biomarkers may serve as a tool for discriminating HT, PA, uPA and bPA. Further studies are needed to support our results.

## Introduction

Primary aldosteronism (PA) represents the most common type of endocrine hypertension and can be found in at least 10% of adult hypertensive patients and in at least 20% of patents with treatment-resistant hypertension [1–3]. In the majority of cases PA does not manifest any pathognomonic symptoms, and pathologic overproduction of aldosterone may develop well before hypertension appears [4]. It is this hormonal disbalance that is connected to significantly worsened cardiovascular outcomes compared to essential (primary) hypertension (HT) – unrelated to blood pressure elevation per se [2]. The diagnostics of PA, especially of its early forms, thus necessitates screening, where the most clinically accepted method is aldosterone-renin ratio (ARR) [3, 5] which has been a subject of repeated criticism [6, 7]. The guideline-recommended screening and confirmatory procedures are time- and effort-demanding, especially when they necessitate adjustment of current antihypertensive medication; the recommended lateralization studies displaying which PA cases are lateralized (or unilateral, uPA), and which are bilateral (bPA), have up to now been invasive; these are some of the reasons why still very few patients with PA are discovered and adequately treated [2, 8, 9].

The complexity and the high cost of traditional clinical work-up of PA has stimulated search for novel markers of the disease and its subtypes (uPA and bPA). Recently, progressive use of machine learning (ML) has been applied to identify optimal PA biomarkers from multiple analyzed parameters [10–13]. In one of these projects a combination of several marker types has been shown to offer adequate distinction between HT and different types of endocrine hypertension, although this algorithm remains to be validated [12]. In other projects, plasma steroid profiling [10] or plasma metabolomics [13] have been used for identification of PA. To our knowledge, no study has yet been found to sufficiently validate a biomarker combination to be clinically applicable to differentiate between bPA and uPA. Continued research of new biomarker types is necessary to be able to differentiate between HT and PA, and between uPA and bPA.

Protein expression in aldosterone producing adenoma (APA) is significantly different compared to nonfunctioning adrenocortical tumors or in the adjacent nontumoral adrenal cortex [14, 15]. Some peripheral blood proteins, including inflammatory markers, are significantly increased in PA, and decrease after initiation of specific PA treatment [16] [17]. Zhou et al. has examined whether markers within the emerging field of peripheral blood proteomics could differentiate bPA from uPA [18], but at present there are currently no other publications describing proteomics as a tool to diagnose PA and its forms according to lateralization.

The aim of this project was to explore proteomic markers in peripheral serum as a possible diagnostic tool in identifying PA and discriminating between uPA and bPA.

## Materials and methods

### Patient sample collection

Our study is based on the retrospective cohort of 88 hypertensive patients that were evaluated for PA in accordance with the current guidelines [5]. Those with confirmed PA underwent unstimulated adrenal vein sampling (AVS) and/or adrenocortical positron emission tomography (PET/CT) utilizing 11C-metomidate (MTO) or a somewhat more specific tracer para-chloro-2-[^18^F]fluoroethyletomidate (18F-CETO) [19].

Three groups were included: 30 patients with HT, 29 patients with bPA and 29 patients with uPA. A total of 88 serum samples were collected. Patients with HT had high-normal renin values and initial aldosterone-renin ratio <50 nmol/mIU. PA patients with an AVS lateralization index (LI) ≥4, or with an adrenocortical PET/CT uptake index ≥1,25 were considered as uPA; individuals with PA who had LI ≤ 3 or an uptake index <1,25 were considered as bPA [5, 20, 21]. See Table S1, Supplement, for baseline clinical characteristics of the study groups and Table S2 för more detailed inclusion and exclusion criteria.

The samples were routinely collected under normokalemia, after pausing of concurrent MRA use for at least 6 weeks, when the diagnosis of either HT or PA vas clarified, but before adrenalectomy or initiation of specific medical treatment for PA by mineralocorticoid receptor antagonists (MRA). Some hypertensive patients in this project were screened for PA during the COVID-19 pandemic, when a temporarily changed routine was in place where the serum was preferentially collected at the same time as the initial screening blood test for PA. In eight patients with bPA the serum was collected after MRA initiation, without a possibility to pause that treatment.

Serum samples from the 88 individuals were included in the analysis by Olink® Explore 384 Cardiometabolic Panel [22]. This cited work describes the method of Proximity Extension Assay (PEA) where each targeted protein is aimed to be bound by two separate antibodies, each of them connected to one of the two corresponding polynucleotide sequences. Once the specific binding between the protein and both of the antibodies has taken place, the mentioned polynucleotides form a double DNA-spiral which is then analyzed by polymerization and NGS-sequencing. Thus, very low concentrations of specific proteins can be detected with a high level of sensitivity and specificity [23].

A total of 85 serum samples (except for serum from 2 uPA patients and 1 HT patient) passed QC warnings, and, subsequently, one sample was identified as an outlier using the standard 1.5 times the interquartile range criterion, and removed, resulting in a total of 84 (29 HT, 29 bPA, and 26 uPA) samples eligible for statistical comparison. Details are described in the data analysis section below.

### Serum extraction and proximity extension assay sample preparation

Serum was extracted from whole blood through centrifugation at 1000 g for 10 minutes and subsequently stored at −70 C. Samples were randomized by a random number generator and placed accordingly by number on a 96-well plate before being sent to Olink Proteomics for proximity extension assay analysis (PEA) [23] using the Olink® Explore 384 Cardiometabolic Panel [22]. The panel above corresponds to analysis of 384 proteins that have earlier been described as markers for cardiovascular and metabolic disturbances (see the list of proteins, as accessed on November 23, 2024 [24]).

### Data analysis

The R-package Olink® Analyze [25] was used for data analysis and statistical comparison. Quality control (QC) was performed by assessing samples for assay warning and QC warning threshold passes, and three samples mentioned above were removed. Following the descriptive analysis, one of the samples (uPA) was classified as an outlier based on relative expression values, and discarded. Five of the proteins in the panel (TIA1, TNF, LDLR, GDF15, PCOLCE) had been marked with assay warnings, and the corresponding results were discarded. The frequency distribution for assessing the normality of the distribution was performed using a histogram and a normal probability plot. Reference median normalization was used for adjusting for potential study origin confounding effects and a t-test comparison was used for confirming successful normalization and confounder removal.

Protein expression values are reported as normalized protein expression (NPX) - which is Olink’s relative protein quantification unit on a log2 scale, standardized in the PEA analysis approach. When using NGS (Next Generation Sequencing) as readout (as in this project), NPX values are produced using a series of calculations based on the number of matched counts. See the reference below for the detailed description of the PEA method and the NPX formula [22].

### Enzyme-linked immunosorbent assay (ELISA)-based protein quantification in serum

The concentration of Coagulation Factor IX (F9) was determined using a commercial assay (Abcam, Cambridge, United Kingdom, ab188393), according to the manufacturer’s instructions. The serum samples were diluted by a factor of 50. In addition, the serum level of HSPB1 was measured with a dilution rate of 1:5 using an ELISA kit (Invitrogen, Waltham, Massachusetts, USA, EHHSPB1) in accordance with the manufacturer’s instructions. Serum samples were diluted 1:5 and utilized to quantify the level of DPP4 through an ELISA assay (Invitrogen, Waltham, Massachusetts, USA, BMS235), following the manufacturer’s protocol. Standard curves and prediction of values have been done in R version 3.6.2.

### Machine learning model

A machine learning model based on multinomial ridge logistic regression with a stratified 5-fold cross-validation ^26^ was used to investigate the likelihood of identifying PA patients and to distinguish their subtypes (bPA and uPA) in a hypertensive patient population. First, we used the proteins identified as statistically significant between the conditions; then, we investigated all 384 proteins in the panel. To reduce overfitting and enhance model performance, we selected the most variable proteins by ranking them according to their standard deviation prior to model training and evaluation. Using the cross-validation approach, all of the studied 84 samples were divided into 5 folds of 16–17 samples, where each of the folds consisted of three relatively even parts of 5–6 samples belonging to each of the study groups of HT, bPA and uPA, respectively. Each fold was used once as a test dataset while the other four folds were used as a training set. The caret R package [26] was used for stratified cross-validation, and the glmnet package [27] for ridge logistic regression.

### Statistics

R-statistics version 3.6.2 was used for calculating statistical differences between groups. A Welch t-test with p-value adjustment for multiple testing using the Benjamini-Hochberg method (q-value) was used for a 2-group comparison, and an ANOVA F-test with a post-hoc test using Tukey p-value adjustment (q-value) was used for a 3-group comparison. A Spearman rank correlation test was used for calculating the correlation between PEA and Elisa validation.

## Results

### Circulating protein expression

**Fig. 1:**
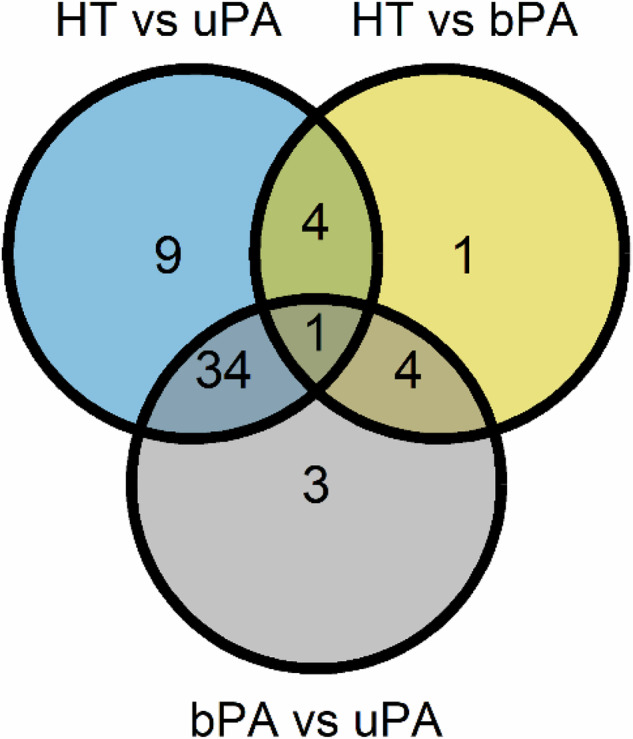
Venn diagram demonstrating the number of significantly different proteins in comparison between the study groups. HT – essential hypertension; PA primary aldosteronism; bPA bilateral PA; uPA unilateral PA

**Fig. 2:**
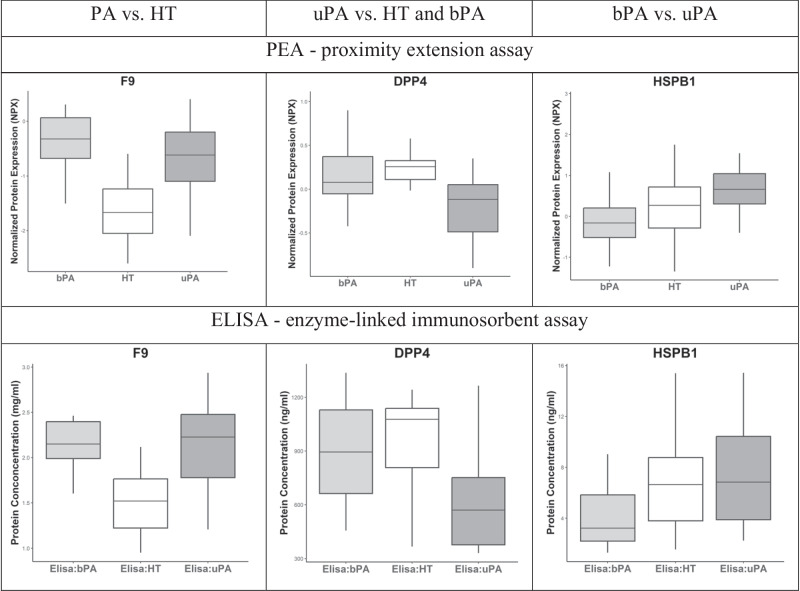
The three serum proteins with maximal differentiating ability between the respective patient groups, analyzed by: A) PEA; and B) ELISA. F9 coagulation factor IX, DPP4 Dipeptidyl peptidase 4, HSPB1 heat shock protein B1, NPX normalized protein expression [22], HT essential hypertension, PA primary aldosteronism, bPA bilateral PA, uPA unilateral PA

**Fig. 3:**
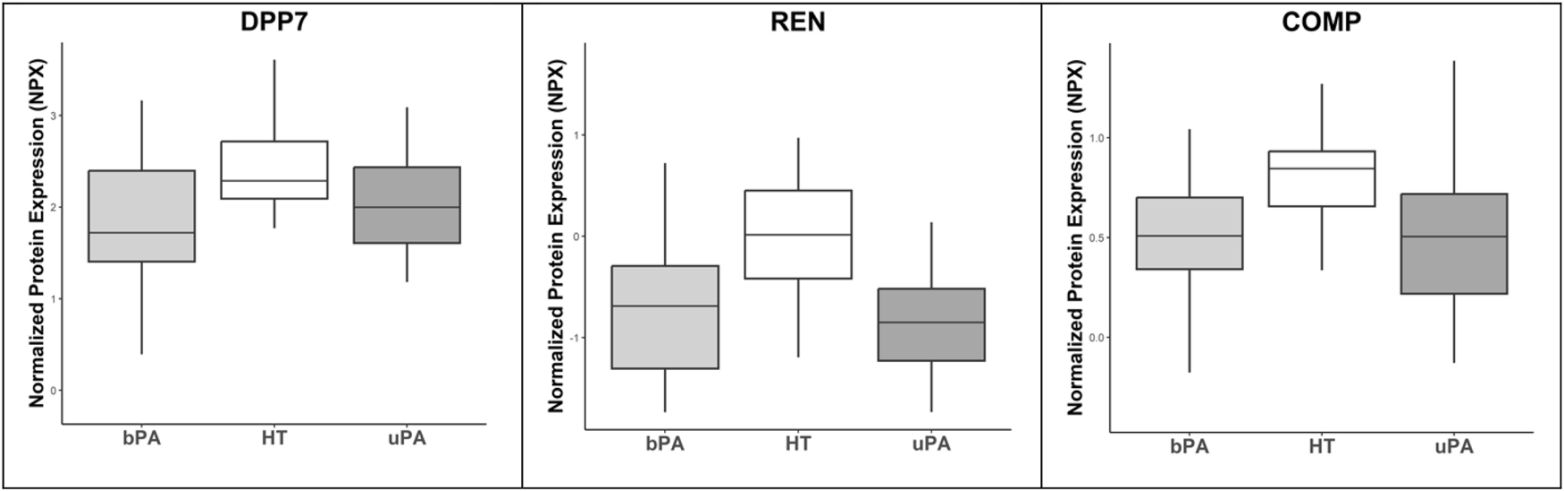
Proteins specifically significantly different between HT and PA (which also includes F9 presented in Fig. 2). DPP7 dipeptidyl peptidase 7, REN renin, COMP cartilage oligomeric matrix protein, NPX normalized protein expression [22], HT essential hypertension, PA primary aldosteronism, bPA bilateral PA, uPA unilateral PA

**Fig. 4:**
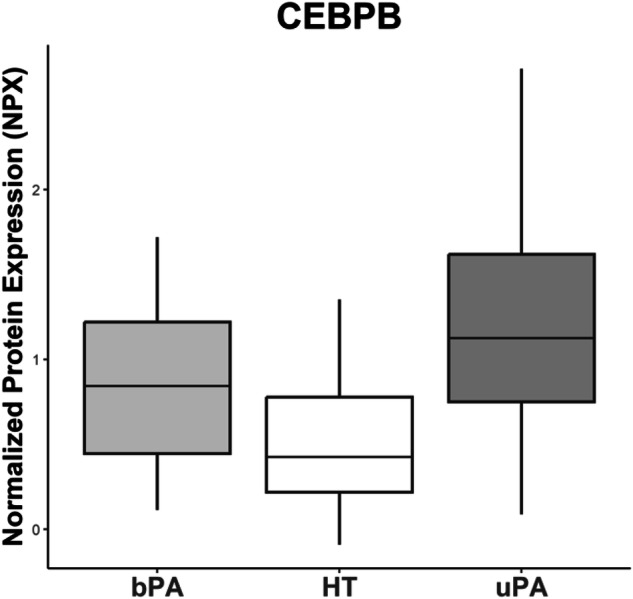
Protein CEBPB (CCAAT/enhancer-binding protein beta) specifically significantly different between HT, bPA and uPA. NPX - normalized protein expression [22], HT essential hypertension, PA primary aldosteronism, bPA bilateral PA, uPA unilateral PA

Comparisons between all of the sample groups of HT, bPA and uPA were performed, which revealed 56 significant (q ≤ 0.05) differentially expressed proteins (see Fig. 1, Table 1, and Table S3 in Supplement). Only four proteins (F9, DPP7, REN, and COMP) were overlapping in both bPA and uPA compared to HT, proving to be unique for comparison PA vs. HT (see Figs. 2 and 3). We proceeded to investigate the underlying differences between the two subsets of PA: uPA and bPA. This disclosed another 42 differentially expressed proteins (q ≤ 0.05) which could be distinguished between these subgroups. Only three of these proteins (ACAN, LACTB2, and HSPB1) were exclusively different when comparing bPA to uPA. Comparing bPA against HT revealed one protein (PAM) to be uniquely differentially expressed, while comparing uPA with HT revealed nine uniquely differentially expressed proteins. Only CEBPB was specifically significantly different between the three study groups of HT, bPA and uPA (see Figs. 1 and 4). See the results of relevant comparisons in Table 1.

**Table 1:** The list of *unique* differentially expressed proteins – specific for the respective comparisons between the study groups according to Fig. 1

Composition of the compared study groups	number of UDEPs^a^	Respective UDEPs^a^
**bPA vs uPA**	3	ACAN, HSPB1, LACTB2
**HT vs uPA & HT vs bPA**^**b**^	4	COMP, DPP7, F9, REN
**HT vs bPA & bPA vs uPA**^**c**^	4	CA13, CCL18, CNTN1, SNAP23
**HT vs uPA & bPA vs uPA**^**d**^	34	APLP1, APOM, AZU1, CA4, CA5A, CCL5, CD14, CDHR5, CEACAM8, CRHR1, DKK3, DPP4, ENPP2, EPHX2, FUCA1, GHRL, GPNMB, IGFBP1, IL1RL1, ITGB2, ITIH3, KIT, LGALS 3, LILRB5, MET, MMP7, MSTN, NPPB, NTproBNP, REG1A, REG3A, SERPINB5, SPON2, TSPAN1
**HT vs uPA & HT vs bPA & bPA vs uPA**^**e**^	1	CEBPB
**HT vs bPA**	1	PAM
**HT vs uPA**	9	ANG, ANGPTL1, CHL1, CRTAC1, DUOX2, LCN2, LTBP2, ST6GAL1, TCN2
**Total**	**56**	

*HT* essential hypertension; *PA* primary aldosteronism; *bPA* bilateral PA; *uPA* unilateral PA

^**a**^UDEPs - unique differentially expressed proteins

^**b**^specifically differentiating HT from all subtypes of PA

^**c**^specifically differentiating bPA from HT or from uPA

^**d**^specifically differentiating uPA from HT or from bPA

^**e**^specifically differentiating the three study groups of HT, bPA and uPA

Out of these results, three proteins were chosen, based on strongest differentiation in expression levels between the sample groups, to be validated by enzyme-linked immunosorbent assay, ELISA (see Fig. 2). Coagulation factor IX (F9) was chosen as a differentiator between PA compared to HT, dipeptidyl peptidase 4 (DPP4) as a differentiator between uPA compared to HT and bPA, and heat shock protein B1 (HSPB1) for comparison of bPA against uPA.

### ELISA-based protein validation

The Olink analysis was validated with specific enzyme-linked immunosorbent assays (ELISAs), and the results from the two analytical methods were compared. For evaluating the levels of F9 and DPP4, 41 of the serum samples (bPA = 14, uPA = 13, and HT = 14) were used, while 35 of the serum samples (bPA = 12, uPA = 10, and HT = 13) were used for determining the level of HSPB1 (see Fig. 2). There was a correlation between the two analytical methods, with correlation for F9: rho = 0.505, p = 0.0007; DPP4: rho = 0.629, p = 0.0001; and HSPB1: rho = 0.824, p = 0.0001 (see Table S4, Supplement, for the respective protein expression values).

### Hypertension differentiation by machine learning

We applied a machine learning model using nine different proteins combined with ARR and could successfully identify 95% of HT, bPA, and uPA samples with an average AUC of 0.991, precision of 0.952, specificity of 0.977, recall (sensitivity) of 0.952, F1 score of 0.952, classification accuracy of 0.928, and Matthew’s correlation coefficient (MCC) of 0.928.

When assessed by class, the model accurately identified all HT cases with perfect AUC, accuracy, precision, sensitivity, specificity, recall (sensitivity), F1 score, and MCC of 1.000.

For bPA, the model had an AUC of 0.985, classification accuracy of 95.2%, precision of 0.931, specificity of 0.964, recall (sensitivity) and F1 score of 0.931, and MCC of 0.895.

**Fig. 5:**
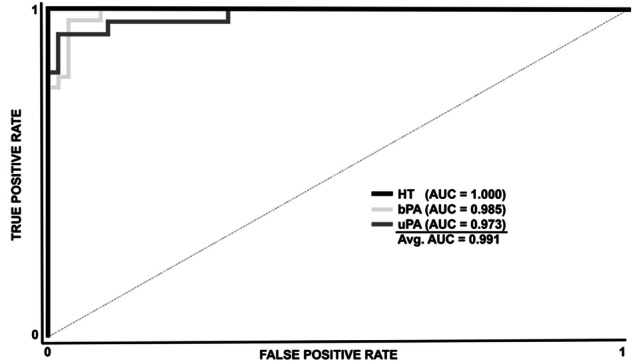
ROC-curve for the used ML model for differentiation of the study groups of HT, bPA and uPA. ML machine learning, AUC area under curve, HT essential hypertension, PA primary aldosteronism, bPA bilateral PA, uPA unilateral PA

For uPA, the AUC was 0.973, accuracy was 95.2%, specificity was 0.966, precision, recall (sensitivity), and F1 score were 0.923, and MCC was 0.889. The algorithm misclassified two bPA cases as uPA and two uPA cases as bPA but properly recognized all HT samples. Figure 5 shows full ROC curves, and Table S5 (Supplement) shows the confusion matrix.

## Discussion

In this study we investigated potential serum protein markers for differentiation between HT, PA and its subtypes. Serum from 29 patients with HT, 26 with uPA, and 29 with bPA was subjected to a proximity extension assay on the cardiometabolic 384 protein panel (Olink® Explore 384). Expression of numerous proteins was significantly different in the studied groups. The three proteins which individually had the strongest differentiating properties between the tested groups of samples (F9, DDP4, HSPB1) were also validated by ELISA.

Significant correlation between the Olink and ELISA results was observed, which was moderate for F9, strong for DPP4 and very strong for HSPB1. The corresponding trends of how the ELISA results follow the Olink (PEA) results for each of these proteins are demonstrated in Fig. 2. That being said, superior sensitivity and specificity of PEA compared to ELISA is well established [28, 29]. However, the antibodies in the applied PEA analysis related to F9, DPP4 and HSPB1 proteins may not be the same as in the used ELISA kits. Based on these conditions, it remains to be proven if ELISA may be applied for clinical analysis of these biomarkers.

The search for circulating protein markers to try to predict both presence of PA and its subtype seems logical. Some studies on PA patients have demonstrated presence of proteomic markers in urinary extracellular vesicles [30, 31] – permitting a degree of differentiation between PA and HT. However, as the extracellular vesicles originate from the cells in direct contact with the urogenital tract [31], these markers may hardly be appropriate for differentiating between different types of adrenal lesions in PA.

Previous studies support the notion that bPA and uPA are different in regard to their genetic background [32], phenotypes [2], as well as long-term prognosis after specific treatment [33]. Recent data indicates that many of the cases of uPA are not a consequence of a classic APA - but of aldosterone producing nodules (APN) or micronodules (APM), often seen in bPA [34, 35]. Still, the optimal treatment method for PA depends on its subtype. Multiple clinical studies including a meta-analysis show that surgically treated cases of uPA have superior outcome compared to medically treated cases of both uPA and bPA [33, 36, 37], even if, lately, better insights have begun to appear on how to efficiently titrate MRA in medically treated cases [38–40]. As efficient treatment for PA exists, and as routine lateralization studies are still invasive, it is important to both optimize diagnostics of PA, and try to find other methods for prediction of uPA / bPA – where the marker analysis may come to play a major role.

Hypothetically, proteomics may represent one of the common denominators of the physiologic status of PA patients - as some proteins may mirror genetic changes in PA or the consequences of extra-adrenal pathologic processes due to abnormally increased aldosterone levels. In the light of the above mentioned mutual differences between uPA and bPA, it is possible that the model presented in our study is based on some inherent pathophysiological variance that indeed exist between HT and PA, as well as between the subtypes of PA. We have searched scientific literature regarding the proteins CEBPB, F9, HSPB1 and DPP4, which, to our knowledge, have not been studied in PA earlier. While here described data does not support direct relation of these tentative markers with the pathogenesis of PA, it may help eventual further studies in that direction.

The CCAAT/enhancer-binding protein beta (CEBPB) is a transcription factor associated with inflammatory response, cytokine regulation, activation of immune cells – as in vascular or bowel inflammatory conditions [41, 42]. In vitro, CEBPB inhibits the gene for the 5-HT1A receptor in the central nervous system, CNS [43]. In adrenals, serotonin locally stimulates aldosterone production [44]. Increased expression of 5-HT1A receptor in the CNS disease, such as depression, is correlated with lower local serotonin levels [43]. In our study we could see significantly higher levels of CEBPB in uPA, lower in bPA and lowest in HT – thus corresponding to the differences in serum aldosterone and ARR between the groups. Even if the serotonin receptors in the adrenal are mostly 5-HT4 and 5-HT7, this hypothetic relation between CEBPB and aldosterone production requires further attention.

Up to 75% of coagulation factor IX (F9) is localized in the vascular subendothelial membrane, connected to collagen type IV [45]. Besides hemostasis, F9 also regulates cell migration and adhesion [46]. Both total mortality and mortality associated to ischemic heart disease is lower in carriers of hemophilia B (and A), especially in older age [47]. It might be that F9 represents in this context an eventual effector (or marker) of endothelial dysfunction, which, however, we cannot support with any other previously published data.

Soluble heat shock protein beta-1 (HSPB1) is produced and secreted by many cell types, including myocytes in the heart and the vasculature. It plays an antiatherogenic role, modulates inflammation and oxidative stress in vascular as well as in cardiac myocytes, stimulates autophagocytosis, suppresses apoptosis; low circulating level of HSPB1 is seen as an emerging marker of atherosclerosis [48, 49].

Dipeptidyl peptidase 4 (DPP4) is produced and secreted by many cell types including endothelium, smooth muscle, immune cells and fat cells, and triggered by hyperglycemia and hypoxia. It cleaves glucagon-like peptide-1, provoking hyperglycemia and insulin resistance. DPP4 is raised in the metabolic syndrome, and stimulates inflammation and proliferation in human vasculature. Circulating DPP4 levels predict the development of type two diabetes, atherosclerosis and hypertension [50].

Prior work on serum biomarkers like plasma steroid profiling [10], and plasma metabolomics [13] has shown promise in differentiating subtypes of PA. Further, Zhou et al., using proteomic profiling, were able to differentiate subtypes using a panel of five serum proteins (APOC3, CD56, CHGA, KRT5, and AZGP1) [18]. Unfortunately, the Olink cardiometabolic profile did not include these proteins and hence we could not confirm or refute these findings.

To assess the potential of finding a biomarker profile that could with high accuracy and precision identify the three patient groups, we applied a machine learning investigation. As we had to do with relatively balanced but small group sizes, we used a stratified 5-fold cross-validation strategy to preserve the proportional representation of each class in both training and test datasets. A 10-fold approach is commonly used; however, with a small dataset, using a 5-fold approach allows us to retain enough samples per group to maintain lower variability, and as we have independent variables as features (proteins), this would still suffice enough to produce a reliable model evaluation [51]. Logistic regression is often acknowledged as adaptable and well suited for most situations and as a good starting point for classification tasks; therefore, it was chosen as our starting point [52, 53]. The risk of overfitting is, of course, present in a small dataset, and the risk for multicollinearity in our multi-class classification is high. Therefore, we applied ridge regularization, as it corrects for possible overfitting by performing coefficient shrinkage [54]. However, an alternative way could be to use a naïve Bayes approach, as this has also been shown to perform well on smaller datasets [55].

The main aim of the study was to examine whether peripheral blood proteomics may provide a marker combination to separate the patients with HT from PA, and also uPA from bPA – the possibility of which we demonstrate. Further studies are needed to validate this hypothesis in detail.

## Limitations

Due to the limited number of available samples from other cohorts of thoroughly clinically investigated patients with proven HT, bPA or uPA, external validation of the study results could not be reached at this stage. Some of the proteins may be differently expressed due to secondary changes not specific for PA. However, some known or possible effects of medicines (such as glucocorticoids, immune suppressors, DPP4 inhibitors, Torasemide) on the protein markers have been controlled for (Table S1). The groups were not balanced in respect to other medicines, even though we have not found any relevant published data supporting their effect on the protein marker expression.

The uneven numbers of cases used for ELISA validation were due to the limited volume of available serum in some patients.

It is within the confines of our project not possible to judge whether the discriminating protein markers were the result of the differences in the adrenal glands of the patients according to the study groups, or whether the markers were related to the specific secondary effects that PA has produced in the target organs and tissues. This paradigm may also be applied to many other marker-related studies of PA. It is known that PA provokes greater target organ damage (TOD) than HT, and that cases of uPA are related to more aggressive primary aldosteronism than those of bPA [2]. As we used a proteomic panel initially conceived to include markers that have been shown to be related to cardiovascular diseases [24], it is possible that the differences in markers between HT and PA, as well as between bPA and uPA were dependent on differences in TOD in these groups.

We present as detailed as possible the appearance of cardiovascular, metabolic and renal diseases in the studied groups (Table S1). It is true that our groups were not balanced in regard to prevalence of these conditions, which was not planned in the initial design of the study. The patients were enrolled retrospectively where the focus was to assure that the cases clearly pertained to one of the three study groups. It was, to the contrary, relevant to expect that the groups of patients with uPA would be more affected by TOD compared with the group of bPA, and that both of these would be more affected by TOD than HT - which trend also seems to be present in our data in Table S1.

## Conclusion

The study explored serum protein biomarkers as a tool for identifying PA in hypertensive patients and discriminating between uPA and bPA. Further studies are needed to support that the proposed diagnostic model may improve and streamline diagnosis of PA, and consequently reduce the need for time and effort-consuming diagnostics, as well as for invasive localizing procedures in subjects with PA. Continued research of proteomic profile as a diagnostic tool in PA is required.

## Supplementary information

Supplementary information

## Data Availability

All the relevant data is presented within the manuscript and the supplement. Other patient related research data are not shared due to privacy or ethical restrictions.

## References

[1] S. Monticone et al., Cardiovascular events and target organ damage in primary aldosteronism compared with essential hypertension: a systematic review and meta-analysis. Lancet Diabetes Endocrinol. **6**(no. 1), 41–50 (2018). 10.1016/S2213-8587(17)30319-4. 29129575 10.1016/S2213-8587(17)30319-4

[2] A. Vaidya, G.L. Hundemer, K. Nanba, W.W. Parksook, J.M. Brown, Primary Aldosteronism: State-of-the-Art Review. Am. J. Hypertens. **35**(no. 12), 967–988 (2022). 10.1093/ajh/hpac079. 35767459 10.1093/ajh/hpac079 PMC9729786

[3] M. Naruse et al., Japan Endocrine Society clinical practice guideline for the diagnosis and management of primary aldosteronism 2021. Endocr. J. **69**(no. 4), 327–359 (2022). 10.1507/endocrj.EJ21-0508. 35418526 10.1507/endocrj.EJ21-0508

[4] A. Vaidya, P. Mulatero, R. Baudrand, G.K. Adler, The expanding spectrum of primary aldosteronism: implications for diagnosis, pathogenesis, and treatment. Endocr. Rev. **39**(no. 6), 1057–1088 (2018). 10.1210/er.2018-00139. 30124805 10.1210/er.2018-00139 PMC6260247

[5] J.W. Funder et al., The management of primary aldosteronism: case detection, diagnosis, and treatment: an endocrine society clinical practice guideline. J. Clin. Endocrinol. Metab. **101**(no. 5), 1889–1916 (2016). 10.1210/jc.2015-4061. 26934393 10.1210/jc.2015-4061

[6] J. Ariens, A.R. Horvath, J. Yang, K.W. Choy, Performance of the aldosterone-to-renin ratio as a screening test for primary aldosteronism in primary care. Endocrine **77**(no. 1), 11–20 (2022). 10.1007/s12020-022-03084-x. 35622194 10.1007/s12020-022-03084-x PMC9242901

[7] A. Hung et al., Performance of the Aldosterone to Renin Ratio as a Screening Test for Primary Aldosteronism. J. Clin. Endocrinol. Metab. **106**(no. 8), 2423–2435 (2021). 10.1210/clinem/dgab348. 34008000 10.1210/clinem/dgab348

[8] J. Funder, Primary aldosteronism. Trends Cardiovasc. Med. **32**(no. 4), 228–233 (2022). 10.1016/j.tcm.2021.03.005. 33775861 10.1016/j.tcm.2021.03.005

[9] E. Gkaniatsa et al., Increasing incidence of primary aldosteronism in western sweden during 3 Decades – Yet an underdiagnosed disorder. J. Clin. Endocrinol. Metab. **106**(no. 9), e3603–e3610 (2021). 10.1210/clinem/dgab327. 33974052 10.1210/clinem/dgab327 PMC8372665

[10] G. Eisenhofer et al., Use of steroid profiling combined with machine learning for identification and subtype classification in primary aldosteronism. JAMA Netw. Open **3**(no. 9), e2016209 (2020). 10.1001/jamanetworkopen.2020.16209. 32990741 10.1001/jamanetworkopen.2020.16209 PMC7525346

[11] Z. Erlic et al., Targeted metabolomics as a tool in discriminating endocrine from primary hypertension. J. Clin. Endocrinol. **106**, 1111–1128 (2021). 10.1210/clinem/dgaa954. 10.1210/clinem/dgaa954 PMC7993566 33382876

[12] P.S. Reel et al., Machine learning for classification of hypertension subtypes using multi-omics: a multi-centre, retrospective, data-driven study. eBioMedicine **84**, 104276 (2022). 10.1016/j.ebiom.2022.104276. 36179553 10.1016/j.ebiom.2022.104276 PMC9520210

[13] J. Song et al., Untargeted metabolomics reveals potential plasma biomarkers for diagnosis of primary aldosteronism using liquid chromatography–mass spectrometry. Biomed. Chromatogr. **38**, e5855 (2024). 10.1002/bmc.5855. 38442715 10.1002/bmc.5855

[14] M.M. Swierczynska et al., Proteomic landscape of aldosterone-producing adenoma. Hypertension **73**(no. 2), 469–480 (2019). 10.1161/HYPERTENSIONAHA.118.11733. 30580688 10.1161/HYPERTENSIONAHA.118.11733

[15] L. Ma et al., Phosphoproteomics reveals the wolframin-calcium axis as an important pathogenic signaling node in primary aldosteronism. Hypertension **80**(no. 5), 995–1010 (2023). 10.1161/HYPERTENSIONAHA.122.20515. 36825503 10.1161/HYPERTENSIONAHA.122.20515

[16] H. Remde et al., The cardiovascular markers copeptin and high-sensitive C-reactive protein decrease following specific therapy for primary aldosteronism. J. Hypertens. **34**(no. 10), 2066–2073 (2016). 10.1097/HJH.0000000000001041. 27442789 10.1097/HJH.0000000000001041

[17] C.A. Carvajal, A. Tapia-Castillo, J.A. Pérez, C.E. Fardella, Serum Alpha-1-acid glycoprotein-1 and urinary extracellular vesicle miR-21-5p as potential biomarkers of primary aldosteronism. Front. Immunol. **12**, 768734 (2021). 10.3389/fimmu.2021.768734. 34804057 10.3389/fimmu.2021.768734 PMC8603108

[18] F. Zhou et al., Targeted multiplex proteomics for the development and validation of biomarkers in primary aldosteronism subtyping. Eur. J. Endocrinol. **191**, 558–569 (2024). 10.1093/ejendo/lvae148. lvae148. 10.1093/ejendo/lvae148 39556467

[19] I. Silins et al., First-in-human evaluation of [18F]CETO: a novel tracer for adrenocortical tumours. Eur. J. Nucl. Med. Mol. Imaging **50**(no. 2), 398–409 (2023). 10.1007/s00259-022-05957-9. 36074157 10.1007/s00259-022-05957-9 PMC9816205

[20] T.J. Burton et al., Evaluation of the sensitivity and specificity of ^11^ C-metomidate positron emission tomography (PET)-CT for lateralizing aldosterone secretion by Conn’s Adenomas. J. Clin. Endocrinol. Metab. **97**(no. 1), 100–109 (2012). 10.1210/jc.2011-1537. 22112805 10.1210/jc.2011-1537

[21] X. Wu et al., [11C]metomidate PET-CT versus adrenal vein sampling for diagnosing surgically curable primary aldosteronism: a prospective, within-patient trial. Nat. Med. **29**(no. 1), 190–202 (2023). 10.1038/s41591-022-02114-5. 36646800 10.1038/s41591-022-02114-5 PMC9873572

[22] L. Wik et al., Proximity extension assay in combination with next-generation sequencing for high-throughput proteome-wide analysis. Mol. Cell. Proteom. **20**, 100168 (2021). 10.1016/j.mcpro.2021.100168. 10.1016/j.mcpro.2021.100168 PMC8633680 34715355

[23] E. Assarsson et al., Homogenous 96-Plex PEA Immunoassay Exhibiting High Sensitivity, Specificity, and Excellent Scalability. PLoS ONE **9**(no. 4), e95192 (2014). 10.1371/journal.pone.0095192. 24755770 10.1371/journal.pone.0095192 PMC3995906

[24] “Olink Explore 3072/384 — Olink®.” Accessed: Nov. 29, 2024. [Online]. Available: https://olink.com/products/olink-explore-3072-384

[25] K. Nevola (kathy-nevola) et al., *OlinkAnalyze: Facilitate Analysis of Proteomic Data from Olink*. (Feb. 22, 2024). Accessed: Apr. 27, 2024. [Online]. Available: https://cran.r-project.org/web/packages/OlinkAnalyze/index.html

[26] M. Kuhn, “caret: Classification and Regression Training.” p. 7.0–1, Oct. 05, (2007). 10.32614/CRAN.package.caret.

[27] J. Friedman et al., “glmnet: Lasso and Elastic-Net Regularized Generalized Linear Models.” p. 4.1-8, (2008). 10.32614/CRAN.package.glmnet

[28] B.I. Arioz, A. Cotuk, E.C. Yaka, S. Genc, Proximity extension assay-based proteomics studies in neurodegenerative disorders and multiple sclerosis. Eur. J. Neurosci. **59**(no. 6), 1348–1358 (2024). 10.1111/ejn.16226. 38105531 10.1111/ejn.16226

[29] A.H. Ren, E.P. Diamandis, V. Kulasingam, Uncovering the depths of the human proteome: antibody-based technologies for ultrasensitive multiplexed protein detection and quantification. Mol. Cell. Proteom. **20**, 100155 (2021). 10.1016/j.mcpro.2021.100155. 10.1016/j.mcpro.2021.100155 PMC9357438 34597790

[30] E.R. Barros et al., Proteomic profile of urinary extracellular vesicles identifies AGP1 as a potential biomarker of primary aldosteronism. Endocrinology **162**(no. 4), bqab032 (2021). 10.1210/endocr/bqab032. 33580265 10.1210/endocr/bqab032

[31] L. Bertolone et al., Proteomic analysis of urinary extracellular vesicles highlights specific signatures for patients with primary aldosteronism. Front. Endocrinol. **14**, 1096441 (2023). 10.3389/fendo.2023.1096441. 10.3389/fendo.2023.1096441 PMC10200877 37223008

[32] U.I. Scholl, Genetics of primary aldosteronism. Hypertension **79**(no. 5), 887–897 (2022). 10.1161/HYPERTENSIONAHA.121.16498. 35139664 10.1161/HYPERTENSIONAHA.121.16498 PMC8997684

[33] M. Reincke, T.A. Williams, True unilateral primary aldosteronism exists, and unilateral adrenalectomy saves lives. Eur. J. Endocrinol. **186**(no. 6), C5–C7 (2022). 10.1530/EJE-22-0123. 35380984 10.1530/EJE-22-0123

[34] M. Iacobone et al., Unilateral adrenal hyperplasia: A novel cause of surgically correctable primary hyperaldosteronism. Surgery **152**(no. 6), 1248–1255 (2012). 10.1016/j.surg.2012.08.042. 23158191 10.1016/j.surg.2012.08.042

[35] T.A. Williams et al., International histopathology consensus for unilateral primary aldosteronism. J. Clin. Endocrinol. Metab. **106**(no. 1), 42–54 (2021). 10.1210/clinem/dgaa484. 32717746 10.1210/clinem/dgaa484 PMC7765663

[36] S.-Y. Chen et al., Cardiovascular outcomes and all-cause mortality in primary aldosteronism after adrenalectomy or mineralocorticoid receptor antagonist treatment: a meta-analysis. Eur. J. Endocrinol. **187**(no. 6), S47–S58 (2022). 10.1530/EJE-22-0375. 36315466 10.1530/EJE-22-0375

[37] N. Qian, J. Xu, Y. Wang, Stroke risks in primary aldosteronism with different treatments: a systematic review and meta-analysis. J. Cardiovasc. Dev. Dis. **9**(no. 9), 300 (2022). 10.3390/jcdd9090300. 36135445 10.3390/jcdd9090300 PMC9505464

[38] G.L. Hundemer, G.C. Curhan, N. Yozamp, M. Wang, A. Vaidya, Cardiometabolic outcomes and mortality in medically treated primary aldosteronism: a retrospective cohort study. Lancet Diabetes Endocrinol. **6**(no. 1), 51–59 (2018). 10.1016/S2213-8587(17)30367-4. 29129576 10.1016/S2213-8587(17)30367-4 PMC5953512

[39] G.L. Hundemer, A.A. Leung, G.A. Kline, J.M. Brown, A.F. Turcu, A. Vaidya, Biomarkers to guide medical therapy in primary aldosteronism. Endocr. Rev. **45**(no. 1), 69–94 (2024). 10.1210/endrev/bnad024. 37439256 10.1210/endrev/bnad024 PMC10765164

[40] J. Yang et al., Outcomes after medical treatment for primary aldosteronism: an international consensus and analysis of treatment response in an international cohort. Lancet Diabetes Endocrinol. **13**(no. 2), 119–133 (2025). 10.1016/S2213-8587(24)00308-5. 39824204 10.1016/S2213-8587(24)00308-5

[41] J.K. Nowak et al., Characterisation of the circulating transcriptomic landscape in inflammatory bowel disease provides evidence for dysregulation of multiple transcription factors including NFE2, SPI1, CEBPB, and IRF2. J. Crohns Colitis **16**(no. 8), 1255–1268 (2022). 10.1093/ecco-jcc/jjac033. 35212366 10.1093/ecco-jcc/jjac033 PMC9426667

[42] Z. Tian, X. Wu, B. Zhang, W. Li, C. Wang, Transcription factor CEBPB mediates intracranial aneurysm rupture by inflammatory and immune response. CNS Neurosci. Ther. **30**(no. 2), e14603 (2024). 10.1111/cns.14603. 38332649 10.1111/cns.14603 PMC10853640

[43] Y.-P. Liu et al., Transcription factor CEBPB inhibits the expression of the human HTR1A by binding to 5′ regulatory region in vitro. Genes **10**(no. 10), 802 (2019). 10.3390/genes10100802. 31614865 10.3390/genes10100802 PMC6827163

[44] E. Louiset, C. Duparc, H. Lefebvre, Role of serotonin in the paracrine control of adrenal steroidogenesis in physiological and pathophysiological conditions. Curr. Opin. Endocr. Metab. Res. **8**, 50–59 (2019). 10.1016/j.coemr.2019.07.003.

[45] D.M. Mann, K.A. Stafford, M. Poon, D. Matino, D.W. Stafford, The Function of extravascular coagulation factor IX in haemostasis. Haemophilia **27**(no. 3), 332–339 (2021). 10.1111/hae.14300. 33780107 10.1111/hae.14300

[46] H. Kitano, A. Mamiya, I. Tomomi, K. Shinichiro, H. Chiaki, Coagulation factor IX regulates cell migration and adhesion in vitro. Cell Biol. Int. **39**(no. 10), 1162–1172 (2015). 10.1002/cbin.10491. 25976981 10.1002/cbin.10491

[47] A. Šrámek, M. Kriek, F. Rosendaal, Decreased mortality of ischaemic heart disease among carriers of haemophilia. Lancet **362**(no. 9381), 351–354 (2003). 10.1016/S0140-6736(03)14021-4. 12907007 10.1016/s0140-6736(03)14021-4

[48] K. Chen et al., HSPB1 regulates autophagy and apoptosis in vascular smooth muscle cells in arteriosclerosis obliterans. Cardiovasc. Ther. **2022**, 1–11 (2022). 10.1155/2022/3889419. 10.1155/2022/3889419 PMC9678445 36474716

[49] Z. Batulan et al., Extracellular release and signaling by heat shock protein 27: role in modifying vascular inflammation. Front. Immunol. **7**, 285 (2016). 10.3389/fimmu.2016.00285. 27507972 10.3389/fimmu.2016.00285 PMC4960997

[50] K.M. Love, Z. Liu, DPP4 activity, hyperinsulinemia, and atherosclerosis. J. Clin. Endocrinol. Metab. **106**(no. 6), 1553–1565 (2021). 10.1210/clinem/dgab078. 33570554 10.1210/clinem/dgab078 PMC8118363

[51] B.G. Marcot, A.M. Hanea, What is an optimal value of k in k-fold cross-validation in discrete Bayesian network analysis?. Comput. Stat. **36**(no. 3), 2009–2031 (2021). 10.1007/s00180-020-00999-9.

[52] M. Meloun, J. Militký, in Statistical data analysis: a practical guide (WPI, Woodhead Publ. India Pvt. Ltd, New Delhi, 2012), Reprinted. in Woodhead Publishing India in materials.

[53] D. Jurafsky, J.H. Martin, in Speech and language processing (Pearson Education, Harlow, 2014), 2. ed.

[54] F. Sohil, M.U. Sohali, J. Shabbir, An introduction to statistical learning with applications in R: by Gareth James, Daniela Witten, Trevor Hastie, and Robert Tibshirani, New York, Springer Science and Business Media, 2013, $41.98, eISBN: 978-1-4614-7137-7. Stat. Theory Relat. Fields **6**(no. 1), 87–87 (2022). 10.1080/24754269.2021.1980261.

[55] A.Y. Ng, M.I. Jordan, in Proceedings of the 15th International Conference on Neural Information Processing Systems: Natural and Synthetic, in NIPS’01 (MIT Press, Cambridge, MA, USA, 2001), pp. 841–848On discriminative vs. generative classifiers: a comparison of logistic regression and naive Bayes.

