# Supplementary material for: Searching for peripheral proteomic markers of primary aldosteronism
Source: Endocrine. 2025 Jun 13;89(3):869–78. doi: 10.1007/s12020-025-04302-y (PMC12370554; doi:10.1007/s12020-025-04302-y)
Supplement: Supplementary file 1 — Supplementary information [file 12020_2025_4302_MOESM1_ESM.docx]

**Supplement**

**Table S1: Baseline clinical characteristics of the study groups.**

Data presented as Mean $\pm$ SD, % or Median (interquartile range).

BMI: body mass index;

SBP: systolic blood pressure;

DBP: diastolic blood pressure;

MRA: mineralocorticoid receptor antagonists;

PAC: plasma aldosterone concentration (under normokalemia and without MRA);

DRC: direct renin concentration (under normokalemia and without MRA);

ARR: aldosterone-renin ratio (under normokalemia and without MRA);

LI: lateralization index;

AVS: adrenal vein sampling;

ACE: angiotensin receptor inhibitor;

ARB: angiotensin receptor blocker; TIA – transitory ischemic attack;

eGFR (mL/min), estimated glomerular filtration rate, MDRD formula (Modification of Diet in Renal Disease Study Group)

DPP4 – Dipeptidyl peptidase 4

**^a^** - renin values below the lowest assessment limit of 1.6 mIU/L were registered as 1.6 mIU/L

**^b^ –** including diuretics (all data on the current groups of medicines are noted at inclusion)

**^c^ -** the other samples from Uppsala University Hospital were stored at – 20 ^0^C.

**^d^ –** relevant as Torasemide inhibits aldosterone synthase as shown in mice [1]

**^e^** - history of acute coronary syndrome / angina / percutaneous coronary intervention

| Baseline characteristics | HT (n = 29) | bPA (n = 29) | uPA (n = 26) |
| --- | --- | --- | --- |
| Sex (% men) | 62 | 38 | 58 |
| Age (years) | $57\pm6$ | 54 $\pm7$ | $53\pm12$ |
| Age when hypertension was first diagnosed  (years) | 47 $\pm$ 10 | 44 $\pm$ 11 | 39 $\pm$ 10 |
| BMI | $30\pm5$ | $29\pm6$ | $31\pm4$ |
| SBP (mmHg) | $140 \pm14$ | $145\pm18$ | 157 $\pm$ 19 |
| DBP (mmHg) | $86\pm9$ | $90\pm11$ | 95 $\pm12$ |
| Initial hypokalemia (%) | 0 | 38 | 19 |
| Serum potassium (mmol/L) | $4.0\pm0.2$ | $3.7\pm$ 0.4 | 3.4 $\pm0.5$ |
| Need for potassium substitution during PA diagnostics (%) | 0 | 38 | 81 |
| Serum creatinine (umol/L) | $72\pm14$ | $73\pm12$ | 73 $\pm17$ |
| eGFR | 82 $\pm$ 9 | 79 $\pm$ 8 | 82 $\pm$ 10 |
| PAC (nmol/L) | 149 (113 – 196 ) | 477 (339 – 733) | 683 (439 – 854) |
| DRC (mIU/L) **^a^** | 30.0 (22.0 – 38.5) | 3.9 (1.7 – 5.2) | 1.8 (1.6 – 4.1) |
| ARR (nmol/mIU) | 5.8 (4.2 – 6.6 ) | 115 (65 – 236) | 271 (172 – 397) |
| Number of antihypertensive medications (n) **^b^** | 2 (1 – 2) | 2 (1 – 3) | 3 (2 – 4) |
| LI at AVS | - | 1.4 (1.1 – 2.0) | 18.3 (6.1 – 21.5) |
| Samples stored at  - 70^0^ C (%) **^c^** | 100 | 100 | 35 |
| Samples taken while on MRA (%) | 0 | 28 | 19 |
| Initial use of MRA (that were paused during PA diagnostics) (%) | 0 | 14 | 23 |
| ACE-inhibitors or ARB (%) | 31 | 66 | 73 |
| Thiazide diuretics (%) | 14 | 35 | 15 |
| Loop diuretics (%) | 0 | 0 | 12 |
| Dihydropyridine calcium blockers (%) | 45 | 48 | 92 |
| Beta-blockers (%) | 14 | 31 | 46 |
| Alpha-1-blockers (%) | 3 | 10 | 31 |
| Verapamil (%) | 0 | 0 | 4 |
| Amilorid (%) | 0 | 3 | 4 |
| Renin inhibitors (%) | 0 | 0 | 0 |
| DPP4 inhibitors (%) | 0 | 0 | 4 |
| Torasemide **^d^** (%) | 0 | 0 | 0 |
| Glucocorticoids (%) | 0 | 0 | 0 |
| Immunodepressants (%) | 0 | 0 | 0 |
| Imidazoline receptor antagonists (%) | 0 | 0 | 0 |
| Ischemic heart disease **^e^** (%) | 1 | 7 | 8 |
| Chronic heart failure (%) | 0 | 0 | 4 |
| Ischemic / hemorrhagic stroke or TIA (%) | 1 | 0 | 8 |
| Diabetes mellitus or glucose intolerance (%) | 10 | 10 | 12 |
| Sleep apnea syndrom (%) | 14 | 10 | 8 |
| Chronic renal failure (%) | 0 | 0 | 4 |
| Atrial fibrillation (%) | 3 | 3 | 23 |
| Hyperlipidemia and/or statin use (%) | 21 | 28 | 8 |

Reference to Table S1:

[1] O. Adam *et al.*, “Inhibition of aldosterone synthase (CYP11B2) by torasemide prevents atrial fibrosis and atrial fibrillation in mice,” *J. Mol. Cell. Cardiol.*, vol. 85, pp. 140–150, Aug. 2015, doi: 10.1016/j.yjmcc.2015.05.019.

**Table S2: Study inclusion and exclusion criteria.**

PA: primary aldosteronism:

DRC: direct renin concentration (under normokalemia and without MRA);

ARR: aldosterone-renin ratio (under normokalemia and without MRA);

LI: lateralization index;

AVS: adrenal vein sampling;

MRA: mineralocorticoid receptor antagonists

| Inclusion: |
| --- |
| - Age $\geq$ 18 years - Possession of the Swedish personal number (permitting access to elective medical care) |
| Inclusion according to the study groups: |
| 1. Essential hypertension:  - Hypertension was initially validated by an existing diagnostic code for hypertension in the patients’ medical journal and/or by $\geq$ 3 documented instances of blood pressure $\geq$ 140/90 at rest - ARR < 50 pmol/mIU at previous screening for PA among consecutive unselected adult primary care patients with hypertension - DRC within upper normal range (15-46 mIU/L) - Absent clinical indications for further investigation concerning other causes of secondary hypertension  1. PA:  - Cases of PA where a well-documented diagnostic work-up for PA was carried out according to the Endocrine Society guidelines, including AVS for assessment of subtype (bPA or uPA)  1. Bilateral PA:  - At AVS, SI $\geq$ 2 and LI $\leq$ 3  1. Unilateral PA:  - At AVS, SI $\geq$ 2 and LI $\geq$ 4 |
| Exclusion: |
| - Pregnancy at the time of PA-diagnostics - Conditions making the risks of diagnostic procedures (saline suppression test and/or pausing of the current MRA treatment ) unacceptably high, such as chronic heart failure with New York Heart Association stage > II, uncontrolled hypertension over 180/110 mmHg in spite of rigorous treatment, severe decompensated chronic illnesses as chronic hepatic, respiratory or renal failure |

**Table S3:** Difference in mean marker content (NPX) between the respective study groups. ANOVA F-test with a post-hoc test using Tukey p-value adjustment (q-value) was used for a 3-group comparison. The corresponding differences are between first-named and second-named groups.

NPX - normalized protein expression [2];

HT – essential hypertension

bPA – bilateral primary aldosteronism

uPA – unilateral primary aldosteronism

| Proteins significantly different between the respective study groups (n = 56) | Compared groups | Difference in mean marker content (NPX) | q-value |
| --- | --- | --- | --- |
| F9 | bPA - HT | 1.17741 | 4.87E-10 |
| APLP1 | HT - uPA | 0.73227 | 9.74E-08 |
| F9 | HT - uPA | -0.92716 | 8.06E-07 |
| CRHR1 | HT - uPA | -0.96822 | 8.54E-07 |
| IGFBP1 | HT - uPA | 1.739444 | 1.35E-06 |
| CA5A | HT - uPA | -1.62534 | 2.51E-06 |
| DPP4 | HT - uPA | 0.436144 | 2.67E-06 |
| DKK3 | HT - uPA | 0.492549 | 4.05E-06 |
| GHRL | HT - uPA | 1.446858 | 7.26E-06 |
| LGALS3 | bPA - uPA | 0.394835 | 1.36E-05 |
| TSPAN1 | bPA - uPA | -1.14494 | 1.47E-05 |
| REG3A | HT - uPA | -0.76352 | 1.76E-05 |
| DPP4 | bPA - uPA | 0.390668 | 2.41E-05 |
| CEBPB | HT - uPA | -0.65381 | 2.59E-05 |
| NTproBNP | bPA - uPA | -1.71344 | 2.93E-05 |
| CRHR1 | bPA - uPA | -0.80811 | 3.45E-05 |
| IL1RL1 | HT - uPA | -0.61431 | 6.37E-05 |
| MMP7 | HT - uPA | -0.28731 | 9.20E-05 |
| CEACAM8 | HT - uPA | -0.89742 | 0.000144334 |
| APLP1 | bPA - uPA | 0.510349 | 0.000160273 |
| CEACAM8 | bPA - uPA | -0.88887 | 0.000167434 |
| CD14 | HT - uPA | 0.350782 | 0.000173047 |
| TSPAN1 | HT - uPA | -0.98759 | 0.000184813 |
| REG3A | bPA - uPA | -0.65231 | 0.00024666 |
| NPPB | bPA - uPA | -1.99906 | 0.000274198 |
| SNAP23 | bPA - uPA | -1.16364 | 0.000279806 |
| CCL5 | HT - uPA | 0.825032 | 0.000301593 |
| KIT | bPA - uPA | 0.323293 | 0.000350929 |
| CNTN1 | bPA - uPA | 0.297257 | 0.000369775 |
| NPPB | HT - uPA | -1.95076 | 0.000389288 |
| NTproBNP | HT - uPA | -1.44608 | 0.000435563 |
| DPP7 | bPA - HT | -0.6509 | 0.000458454 |
| IL1RL1 | bPA - uPA | -0.53661 | 0.000499322 |
| FUCA1 | HT - uPA | 0.763736 | 0.000519566 |
| SERPINB5 | bPA - uPA | 0.706863 | 0.00052977 |
| ITGB2 | HT - uPA | 0.291412 | 0.000695971 |
| CCL5 | bPA - uPA | 0.764426 | 0.000851396 |
| CCL18 | bPA - uPA | -0.54408 | 0.000862169 |
| REN | HT - uPA | 0.7649 | 0.000884181 |
| GHRL | bPA - uPA | 1.069796 | 0.000953225 |
| ITIH3 | bPA - uPA | 0.637308 | 0.001060038 |
| ANGPTL1 | HT - uPA | -0.26686 | 0.001328205 |
| REG1A | HT - uPA | -0.59483 | 0.001362971 |
| CA4 | bPA - uPA | 0.237496 | 0.001364818 |
| CRTAC1 | HT - uPA | 0.319767 | 0.001426485 |
| EPHX2 | HT - uPA | -0.63412 | 0.00157428 |
| FUCA1 | bPA - uPA | 0.696387 | 0.001663077 |
| CA5A | bPA - uPA | -1.08079 | 0.0018542 |
| ST6GAL1 | HT - uPA | 0.342959 | 0.001895625 |
| SPON2 | HT - uPA | 0.322712 | 0.002258098 |
| PAM | bPA - HT | -0.22512 | 0.002329573 |
| COMP | bPA - HT | -0.30632 | 0.002352937 |
| DUOX2 | HT - uPA | -0.75499 | 0.002407371 |
| CD14 | bPA - uPA | 0.283406 | 0.002753358 |
| CA13 | bPA - uPA | -0.89873 | 0.00289388 |
| ACAN | bPA - uPA | -0.28432 | 0.003020015 |
| ANG | HT - uPA | -0.33973 | 0.003383468 |
| CNTN1 | bPA - HT | 0.239476 | 0.003639136 |
| CDHR5 | HT - uPA | -0.31908 | 0.003661341 |
| LILRB5 | bPA - uPA | 0.685187 | 0.003883198 |
| IGFBP1 | bPA - uPA | 1.049265 | 0.003942426 |
| DKK3 | bPA - uPA | 0.310598 | 0.004188775 |
| LTBP2 | HT - uPA | 0.343754 | 0.004246262 |
| APOM | HT - uPA | 0.25424 | 0.004499857 |
| MSTN | HT - uPA | -0.47361 | 0.004572539 |
| LACTB2 | bPA - uPA | -0.40263 | 0.004615954 |
| CA13 | bPA - HT | -0.83161 | 0.004820659 |
| TCN2 | HT - uPA | -0.30707 | 0.004851314 |
| HSPB1 | bPA - uPA | -0.70987 | 0.004869642 |
| KIT | HT - uPA | 0.258706 | 0.004976686 |
| LCN2 | HT - uPA | -0.48416 | 0.005145527 |
| MET | HT - uPA | 0.188384 | 0.005264672 |
| ENPP2 | bPA - uPA | 0.271176 | 0.005484243 |
| AZU1 | HT - uPA | -0.82779 | 0.005564402 |
| CHL1 | HT - uPA | 0.203627 | 0.005709942 |
| EPHX2 | bPA - uPA | -0.54846 | 0.00714736 |
| COMP | HT - uPA | 0.281404 | 0.007344425 |
| ITGB2 | bPA - uPA | 0.23385 | 0.00777307 |
| SPON2 | bPA - uPA | 0.284792 | 0.007926328 |
| LGALS3 | HT - uPA | 0.244976 | 0.008634163 |
| REN | bPA - HT | -0.59221 | 0.009808031 |
| GPNMB | bPA - uPA | 0.212246 | 0.011662046 |
| GPNMB | HT - uPA | 0.211426 | 0.012043174 |
| SNAP23 | bPA - HT | -0.80701 | 0.012180604 |
| ENPP2 | HT - uPA | 0.239897 | 0.015895986 |
| CCL18 | bPA - HT | -0.39339 | 0.016850855 |
| ITIH3 | HT - uPA | 0.479518 | 0.017460929 |
| APOM | bPA - uPA | 0.216902 | 0.017986948 |
| LILRB5 | HT - uPA | 0.57419 | 0.018442188 |
| MMP7 | bPA - uPA | -0.17594 | 0.022614206 |
| SERPINB5 | HT - uPA | 0.483032 | 0.023902613 |
| MSTN | bPA - uPA | -0.38447 | 0.026056299 |
| REG1A | bPA - uPA | -0.42109 | 0.031124948 |
| AZU1 | bPA - uPA | -0.64415 | 0.039158186 |
| CDHR5 | bPA - uPA | -0.23768 | 0.03956655 |
| MET | bPA - uPA | 0.143753 | 0.042598468 |
| CA4 | HT - uPA | 0.159038 | 0.04411364 |
| CEBPB | bPA - uPA | -0.33407 | 0.045593602 |
| DPP7 | HT - uPA | 0.405957 | 0.048003884 |
| CEBPB | bPA - HT | 0.319738 | 0.049974175 |

Reference to Table S3:

[2] L. Wik *et al.*, “Proximity Extension Assay in Combination with Next-Generation Sequencing for High-throughput Proteome-wide Analysis,” *Mol. Cell. Proteomics*, vol. 20, p. 100168, 2021, doi: 10.1016/j.mcpro.2021.100168.

**Table S4:** Expression of the serum proteins HSPB1, F9 and DPP4 in the study samples, analyzed by both ELISA and PEA.

HSPB1 – heat shock protein B1

F9 – coagulation factor IX

DPP4 - Dipeptidyl peptidase 4

PEA – proximity extension assay

NPX - normalized protein expression [2]

ELISA - enzyme-linked immunosorbent assay

HT – essential hypertension

bPA – bilateral primary aldosteronism

uPA – unilateral primary aldosteronism

| **HSPB1** | | | |
| --- | --- | --- | --- |
| **Sample** | **Condition** | **Concentration by ELISA (ng/ml)** | **Protein content by PEA**  **(NPX)** |
| OS-26 | bPA | 5.5594965 | -0.0567 |
| OS-61 | bPA | 6.699489 | -0.0238 |
| OS-30 | bPA | 1.3098505 | -0.1512 |
| OS-54 | bPA | 2.8390875 | -0.365 |
| OS-70 | bPA | 4.152401 | -0.1504 |
| OS-88 | bPA | 2.396215 | -0.3559 |
| OS-19 | bPA | 9.033721 | 1.7074 |
| OS-1 | bPA | 1.8542175 | -0.1618 |
| OS-24 | bPA | 3.629452 | 0.2077 |
| OS-16 | bPA | 6.6796725 | 1.0168 |
| OS-76 | bPA | 2.3386085 | -0.5572 |
| OS-57 | bPA | 1.382556 | -0.6238 |
| OS-66 | HT | 15.399241 | 0.4468 |
| OS-84 | HT | 10.476779 | 1.7489 |
| OS-77 | HT | 10.012356 | 1.4883 |
| OS-9 | HT | 2.758241 | -0.5701 |
| OS-50 | HT | 1.557752 | -1.3474 |
| OS-18 | HT | 6.639988 | 0.214 |
| OS-46 | HT | 4.0122955 | 0.2934 |
| OS-40 | HT | 8.6284645 | 1.4718 |
| OS-39 | HT | 8.776326 | 1.3571 |
| OS-87 | HT | 3.7986995 | -0.6138 |
| OS-33 | HT | 2.1622095 | -0.8584 |
| OS-62 | HT | 6.85741 | 0.6469 |
| OS-48 | HT | 5.069557 | 0.27 |
| OS-82 | uPA | 4.652446 | -0.1152 |
| OS-38 | uPA | 9.7064765 | 1.0266 |
| OS-85 | uPA | 11.954913 | 1.0065 |
| OS-72 | uPA | 6.4605275 | 1.385 |
| OS-22 | uPA | 2.251108 | -0.4023 |
| OS-4 | uPA | 7.209 | 1.05062 |
| OS-23 | uPA | 15.433459 | 0.51632 |
| OS-17 | uPA | 2.8924865 | 0.32512 |
| OS-58 | uPA | 10.672147 | 2.83752 |
| OS-63 | uPA | 3.629452 | 0.74522 |
|  |  |  |  |
| **F9** | | | |
| **Sample** | **Condition** | **Concentration by ELISA (mg/ml)** | **Protein content by PEA**  **(NPX)** |
| OS-86 | bPA | 2.0053745 | -0.325 |
| OS-26 | bPA | 1.877721 | -1.181 |
| OS-61 | bPA | 2.155045 | -0.6341 |
| OS-30 | bPA | 1.9846635 | 0.2286 |
| OS-54 | bPA | 2.3482905 | -0.3443 |
| OS-47 | bPA | 2.462512 | -0.486 |
| OS-70 | bPA | 2.060233 | -0.7263 |
| OS-88 | bPA | 2.144214 | -0.6769 |
| OS-19 | bPA | 2.4028625 | -0.6915 |
| OS-1 | bPA | 2.397618 | -0.1295 |
| OS-24 | bPA | 2.396123 | -0.3343 |
| OS-16 | bPA | 2.450091 | 0.1061 |
| OS-76 | bPA | 1.2428345 | -0.0573 |
| OS-57 | bPA | 1.602973 | 0.065 |
| OS-66 | HT | 1.1144875 | -1.9349 |
| OS-84 | HT | 1.0839415 | -1.7882 |
| OS-3 | HT | 1.546937 | -2.1 |
| OS-77 | HT | 1.728967 | -1.0693 |
| OS-9 | HT | 2.117156 | -0.5942 |
| OS-50 | HT | 1.6302185 | -0.9785 |
| OS-18 | HT | 1.343695 | -2.3055 |
| OS-46 | HT | 1.7774195 | -0.8794 |
| OS-40 | HT | 2.035211 | -0.9856 |
| OS-39 | HT | 1.4941465 | -1.6665 |
| OS-87 | HT | 1.1843415 | -2.1945 |
| OS-33 | HT | 0.9520445 | -2.2139 |
| OS-62 | HT | 1.437173 | -1.9315 |
| OS-48 | HT | 1.892155 | -1.3413 |
| OS-82 | uPA | 1.8203145 | -0.6177 |
| OS-38 | uPA | 2.7718165 | -0.2115 |
| OS-85 | uPA | 1.565816 | -1.9703 |
| OS-72 | uPA | 1.778913 | -0.6823 |
| OS-22 | uPA | 2.4773895 | 0.4043 |
| OS-41 | uPA | 1.763063 | 0.3648 |
| OS-64 | uPA | 2.095482 | 0.216 |
| OS-68 | uPA | 1.2078315 | 0.353 |
| OS-4 | uPA | 2.937305 | 0.2046 |
| OS-23 | uPA | 2.7253045 | -0.9314 |
| OS-17 | uPA | 2.3504665 | -1.0018 |
| OS-58 | uPA | 2.227717 | -1.6854 |
| OS-63 | uPA | 2.3425035 | -0.4997 |
|  |  |  |  |
| **DPP4** | | | |
| **Sample** | **Condition** | **Concentration by ELISA (ng/ml)** | **Protein content by PEA**  **(NPX)** |
| OS-86 | bPA | 1334.17045 | 0.6821 |
| OS-26 | bPA | 876.16825 | -0.0515 |
| OS-61 | bPA | 1336.7441 | 0.5795 |
| OS-30 | bPA | 1158.37555 | 0.4509 |
| OS-54 | bPA | 579.05985 | 0.2739 |
| OS-47 | bPA | 556.1126 | -0.0457 |
| OS-70 | bPA | 789.58485 | -0.1324 |
| OS-88 | bPA | 913.0321 | 0.2643 |
| OS-19 | bPA | 730.1401 | -0.1678 |
| OS-1 | bPA | 457.20675 | 0.0044 |
| OS-24 | bPA | 640.7383 | 0.1093 |
| OS-16 | bPA | 1078.3166 | 1.0203 |
| OS-76 | bPA | 1147.10045 | 0.0388 |
| OS-57 | bPA | 1023.07625 | 0.2717 |
| OS-66 | HT | 888.7866 | 0.0989 |
| OS-84 | HT | 872.55695 | 0.1562 |
| OS-3 | HT | 1242.1927 | 0.3176 |
| OS-77 | HT | 446.5582 | 0.2347 |
| OS-9 | HT | 1121.0363 | 0.0406 |
| OS-50 | HT | 367.35845 | 0.2226 |
| OS-18 | HT | 1144.49685 | 0.3273 |
| OS-46 | HT | 785.8984 | 0.2733 |
| OS-40 | HT | 570.11025 | 0.2667 |
| OS-39 | HT | 1154.9075 | 0.3379 |
| OS-87 | HT | 1053.8126 | 0.655 |
| OS-33 | HT | 1229.2632 | 0.7824 |
| OS-62 | HT | 1100.13675 | 0.4321 |
| OS-48 | HT | 1114.07475 | 0.1903 |
| OS-82 | uPA | 354.79785 | -0.5496 |
| OS-38 | uPA | 1186.94905 | 0.3518 |
| OS-85 | uPA | 570.11025 | 0.0477 |
| OS-72 | uPA | 543.02805 | 0.0773 |
| OS-22 | uPA | 549.0777 | -0.5092 |
| OS-41 | uPA | 721.6961 | 0.2237 |
| OS-64 | uPA | 991.3255 | 0.2594 |
| OS-68 | uPA | 377.5337 | 0.0361 |
| OS-4 **^a^** | uPA | 331.5808 | -0.4295 |
| OS-23 | uPA | 375.28015 | -0.3064 |
| OS-17 | uPA | 703.7949 | -0.4834 |
| OS-58 | uPA | 1264.5806 | -0.0012 |
| OS-63 | uPA | 751.62055 | -0.4337 |

**^a^** – this patient was the only one of the study subjects taking DPP4 inhibitor

**Table S5: Differentiation of HT, bPA and uPA by logistic regression.**

HT – essential hypertension

bPA – bilateral primary aldosteronism

uPA – unilateral primary aldosteronism

| Confusion matrix for logistic regression | | | | | |
| --- | --- | --- | --- | --- | --- |
|  | | Predicted: | | | |
|  |  | bPA | HT | uPA | Total |
| Actual: | bPA | 27 | 0 | 2 | 29 |
|  | HT | 0 | 29 | 0 | 29 |
|  | uPA | 2 | 0 | 24 | 26 |
|  | Total | 29 | 29 | 26 | 84 |
